# Effects of Gas Layer Thickness on Capillary Interactions at Superhydrophobic Surfaces

**DOI:** 10.1021/acs.langmuir.3c03709

**Published:** 2024-02-22

**Authors:** Mimmi Eriksson, Per M. Claesson, Mikael Järn, Viveca Wallqvist, Mikko Tuominen, Michael Kappl, Hannu Teisala, Doris Vollmer, Joachim Schoelkopf, Patrick A.C. Gane, Jyrki M. Mäkelä, Agne Swerin

**Affiliations:** † Materials and Surface Design, RISE Research Institutes of Sweden, SE-11486 Stockholm, Sweden; ‡ Department of Chemistry, Division of Surface and Corrosion Science, KTH Royal Institute of Technology, SE-10044 Stockholm, Sweden; § Department of Physics at Interfaces, Max Planck Institute for Polymer Research, D-55128 Mainz, Germany; ∥ Omya International AG, CH-4665 Oftringen, Switzerland; ⊥ School of Chemical Engineering, Department of Bioproducts and Biosystems, Aalto University, FI-00076 Aalto, Finland; # Faculty of Technology and Metallurgy, University of Belgrade, Karnegijeva 4, Belgrade 11000, Serbia; ∇ Physics Unit, Aerosol Physics Laboratory, Tampere University, Tampere FI-33014, Finland; ○ Department of Engineering and Chemical Sciences, Karlstad University, SE-651 88 Karlstad, Sweden; ◆ CR Colloidal Resource AB, Naturvetarvägen 14, SE-22362 Lund, Sweden; ¶ Nordtreat Oy, Mestarintie 11, FI-01730 Vantaa, Finland; & Amcor Flexibles Valkeakoski Oy, Niementie 161, P.O. Box 70, 37601 Valkeakoski, Finland

## Abstract

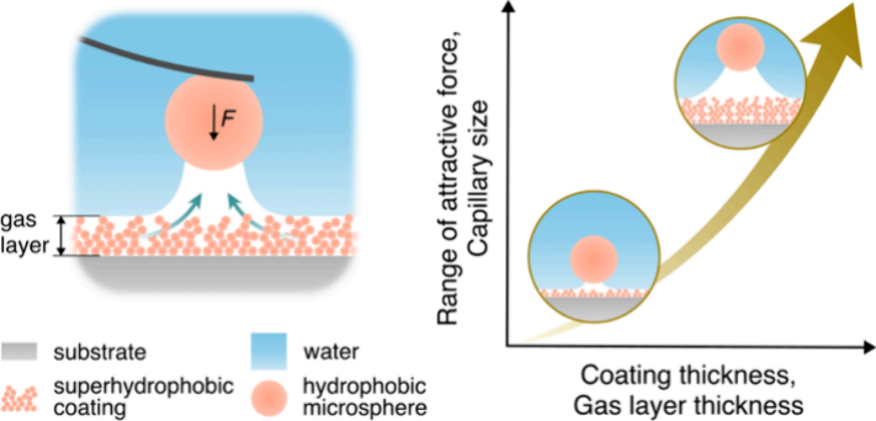

Strongly attractive forces act between superhydrophobic surfaces across water due to the formation of a bridging gas capillary. Upon separation, the attraction can range up to tens of micrometers as the gas capillary grows, while gas molecules accumulate in the capillary. We argue that most of these molecules come from the pre-existing gaseous layer found at and within the superhydrophobic coating. In this study, we investigate how the capillary size and the resulting capillary forces are affected by the thickness of the gaseous layer. To this end, we prepared superhydrophobic coatings with different thicknesses by utilizing different numbers of coating cycles of a liquid flame spraying technique. Laser scanning confocal microscopy confirmed an increase in gas layer thickness with an increasing number of coating cycles. Force measurements between such coatings and a hydrophobic colloidal probe revealed attractive forces caused by bridging gas capillaries, and both the capillary size and the range of attraction increased with increasing thickness of the pre-existing gas layer. Hence, our data suggest that the amount of available gas at and in the superhydrophobic coating determines the force range and capillary growth.

## Introduction

Superhydrophobic surfaces are characterized by high apparent water contact angles (typically >150°).^1,2^ In addition, the roll off angle is small (typically <5–10°). Superhydrophobicity can most easily be achieved for a low energy surface having roughness features on both micro- and nanoscales. The latter was addressed in work to design more mechanically robust superhydrophobic surfaces.^3^ There are many potential applications using superhydrophobicity,^4^ such as for antifouling, but there remain, nonetheless, some practical drawbacks that may limit its applicability. Measurements of interactions between superhydrophobic surfaces are important for many practical applications and are particularly relevant, for example, to where drop-to-surface or particle-to-surface adhesion applies.^5,6^ The latter primarily capture capillary forces established across water, which have been shown to be strongly attractive with a range that may extend to micrometers as the surfaces are pulled apart.^7−11^ These micrometer-ranged attractive forces are due to the formation of a gas (air or vapor) capillary bridge between the two surfaces. During separation, the capillary volume first increases, and at larger separations, it decreases, which gives rise to a characteristic shape of the measured force–distance curves.^12,13^ We have elucidated the formation and evolution of gas capillaries from microscopy images taken during force measurements between a hydrophobic colloidal probe and superhydrophobic coating, quantifying changes in contact angles and capillary volume with surface separation^11^ and, recently, extended the studies to superamphiphobic surfaces.^14^

Initially, the capillary growth during the separation process may be influenced by the diffusion of gases dissolved in the aqueous phase into the capillary. However, as similarly shaped force–distance curves have been observed in both degassed^9^ and normal aerated water,^11^ it is likely that capillary growth is mainly caused by transport of gas present in and between the rough and porous features of the superhydrophobic surface into the capillary. Water on a superhydrophobic surface resides on top of the surface features with a gaseous layer entrapped below, which is known as the Cassie–Baxter state.^15^ The pre-existing gaseous layer can act as a reservoir and facilitate capillary growth. However, it is unclear whether the interactions and capillary growth depend on the amount of gas present in this gaseous layer or whether vaporization of water or dissolved gases alone is sufficient. The properties at the three-phase contact line (TPCL) in the Cassie–Baxter state of superhydrophobicity are discussed in recent work, based on thermodynamics^16^ and force analyses.^17^ Experimental techniques combining force measurements and gas capillary imaging could support the development of theoretical approaches.

In the present study, we investigate how different coating thicknesses, and thereby different thicknesses of the gaseous layer residing on the surface and within near-surface pores of superhydrophobic coatings, influence interactions and gas capillary shape and size. A thermal aerosol-assisted liquid flame spray (LFS) coating method^18^ was used to prepare superhydrophobic surfaces. In LFS, organometallic molecules in a liquid precursor solution are atomized by means of a high-temperature hydrogen–oxygen flame.^19^ The organometallic molecules react in the flame to form nanoparticles. As a substrate is passed through the flame, nanoparticles will deposit and form a porous nanostructured coating that is highly suited for achieving superhydrophobicity.^18,20,21^ By applying a different number of subsequent coating cycles, samples with different coating layer thicknesses are prepared while maintaining similar hydrophobicity.

The interaction forces between a hydrophobic microsphere and the superhydrophobic surfaces were measured by using a colloidal probe atomic force microscopy (AFM) technique. Due to the large range of forces, a piezo with very large range was employed. Gas capillaries were imaged during force measurements using laser scanning confocal microscopy (LSCM) that allowed the local wetting of superhydrophobic surfaces to be elucidated with microscale resolution.^22−25^ From these images, we visualize and quantify the evolution of the capillary and the contact angles during the force measurement. At the same time, the attractive forces as a function of the surface porous coating layer thickness were investigated, and correlation was sought with respect to the observed gas capillaries formed. The AFM-LSCM combination allows a comparison to be made between the free energy change calculated from force–distance curves with contributions calculated from the gas capillary menisci shapes. These contributions, in turn, would stem from surface tension area work, pressure–volume work, and the TPCLs.

## Experimental Section

### Sample Preparation

High-precision cover glass (No.1.5H, thickness 170 ± 5 μm, Carl Roth GmbH) was used as a support for superhydrophobic coatings of different thicknesses, as detailed by Teisala et al.^18^

In the first step, LFS was used for applying a titanium dioxide–silicon dioxide nanostructured coating. The combustion gases and their flow rates were hydrogen (50 L min^–1^) and oxygen (15 L min^–1^), which achieved the needed high-temperature flame. The isopropanol precursor solution contained 50 mg mL^–1^ tetraethyl orthosilicate (98%, Alfa Aesar) and titanium(IV)isopropoxide (97%, Alfa Aesar) with a Ti/Si weight ratio of 99:1. The injection flow rate into the flame was 12 mL min^–1^. The substrates were allowed to pass through the flame at a distance of 6 cm from the burner face. The velocity of the substrates through the flame was kept at 0.8 m s^–1^. Different coating layer thicknesses were prepared by allowing the sample to pass through the flame between 1 and 5 times.

In the next step, the surface energy of the nanostructured coatings was reduced by utilizing fluorosilane and chemical vapor deposition (CVD). We note that the photocatalytic activity of titanium dioxide may cause degradation of the fluorosilane. To avoid this, a thin layer of silicon dioxide was grown on the surface prior to the silane treatment. The thin protective silicon dioxide layer was grown by utilizing a gas-phase Stöber-like reaction in a closed desiccator containing the samples as well as two open vials with ammonia (3 mL, 25%, VWR Chemicals) and tetraethyl orthosilicate (3 mL, 98%, Sigma-Aldrich). The reaction was carried out at room temperature and atmospheric pressure and allowed to proceed for 4 h. The surface was then cleaned and activated by oxygen plasma (Femto low-pressure plasma system, Diener Electronic) at a power of 300 W for 10 min. Fluorosilanization of the activated surface was carried out at a reduced pressure of 100 mbar for 2 h. The reaction vessel was a desiccator containing the samples and 100 μL of 1*H*,1*H*,2*H*,2*H* perfluorooctyl-trichlorosilane (97%, Sigma-Aldrich). Unreacted silane was removed from the surfaces by placing them in a vacuum oven at 60 °C for 2 h.

Glass colloidal probes with a diameter in the range 10–40 μm (Polysciences Inc.) were attached to tipless cantilevers (NSC35/tipless/Cr–Au, Mikromasch) using two-component glue (Epoxy Rapid, Bostik) together with a micromanipulator placed under an optical microscope. The colloidal probes were, after attachment to cantilevers, surface-modified with fluorosilane in the same manner as for the superhydrophobic coatings. For determination of the macroscopic contact angles of the modified particle surface, chemically similar flat samples were prepared by fluorosilanization of microscope glass slides. The cantilever spring constant *k*_*z*_ was determined using the Sader method^26^ to be *k*_*z*_ = 19 N m^–1^. To avoid random variations, the same cantilever and probe particle were used in all experiments.

### Surface Characterization

Scanning electron microscopy (SEM) was utilized for determining the morphology of the nanostructured LFS coatings. To reduce surface charging, the samples were sputter-coated with a thin layer of gold prior to SEM imaging. Top-view and cross-sectional images of the coatings were recorded using an FEI Quanta 250 FEG SEM and a Zeiss Sigma 300 VP SEM, respectively. The colloidal probe diameter was determined from low-vacuum SEM (LV-SEM) images recorded with the FEI Quanta 250 FEG SEM instrument at a pressure of 70 Pa. No surface coating was needed in this case.

Macroscopic water contact angles (CA) were measured by using purified water (Milli-Q, Type 1) and drop shape analysis with a goniometer (OCA40, Dataphysics GmbH). Advancing contact angles were measured by increasing the drop volume (1 μL s^–1^) from 5 to 25 μL, while receding contact angles were obtained by decreasing the volume back to <1 μL. The CAs were determined with the tangent fitting method in the SCA 20 software (Dataphysics GmbH) and reported as the mean values when the droplet advanced or receded over the surface. Five measurements at different positions on the coating surface were performed, and the results are presented as mean ± standard deviations. Roll-off angles (RA) were determined for 10 μL drops as the sample was tilted at a rate of 0.3° s^–1^. The measurement was stopped when the water droplet was rolled off the coating surface. Five droplets at different positions were analyzed for each sample.

Water droplets stationary on the superhydrophobic coatings were imaged by using an inverted LSCM (Leica TCS SP8 SMD, Leica Microsystems) with an HC PL APO CS2 40×/1.10 water objective.

### Laser Scanning Confocal Microscopy Combined with Colloidal Probe Atomic Force Microscopy

The instrument utilized for imaging and force measurements is specially designed and consists of an inverted LSCM coupled with a JPK NanoWizard AFM (JPK Instruments AG).^11,27,28^

For surfaces showing attractive forces exceeding 15 μm, the force range exceeded the range of the internal AFM piezo scanner. To record such long-range forces, the AFM head was moved toward and away from the surface with a speed of 0.20–0.22 μm s^–1^ by means of an external piezo (Physik Instrumente P-622.ZCL piezo stage with 250 μm of closed loop operation). The tip position was determined from the piezo displacement, while the cantilever bending was determined with AFM in a normal manner.

The confocal microscope utilized a 473 nm laser (Cobolt Blues 25 mW) and a 40×/0.95 dry objective (Olympus). The water phase was visualized by adding a water-soluble fluorescent dye (Atto 488, Atto-tec GmbH) at a low concentration (10 mg L^–1^). The signal from the aqueous phase and the light reflection from the interfaces were detected simultaneously using two different detectors. The laser was scanned along one line parallel to the surface and at different heights to render a 2D cross-sectional image. The final image was obtained as the average of 32 line scans. Confocal images were recorded at an acquisition rate of 1 frame s^–1^.

## Results and Discussion

### Morphology and Layer Thickness of the Nanostructured Coatings

**Figure 1:**
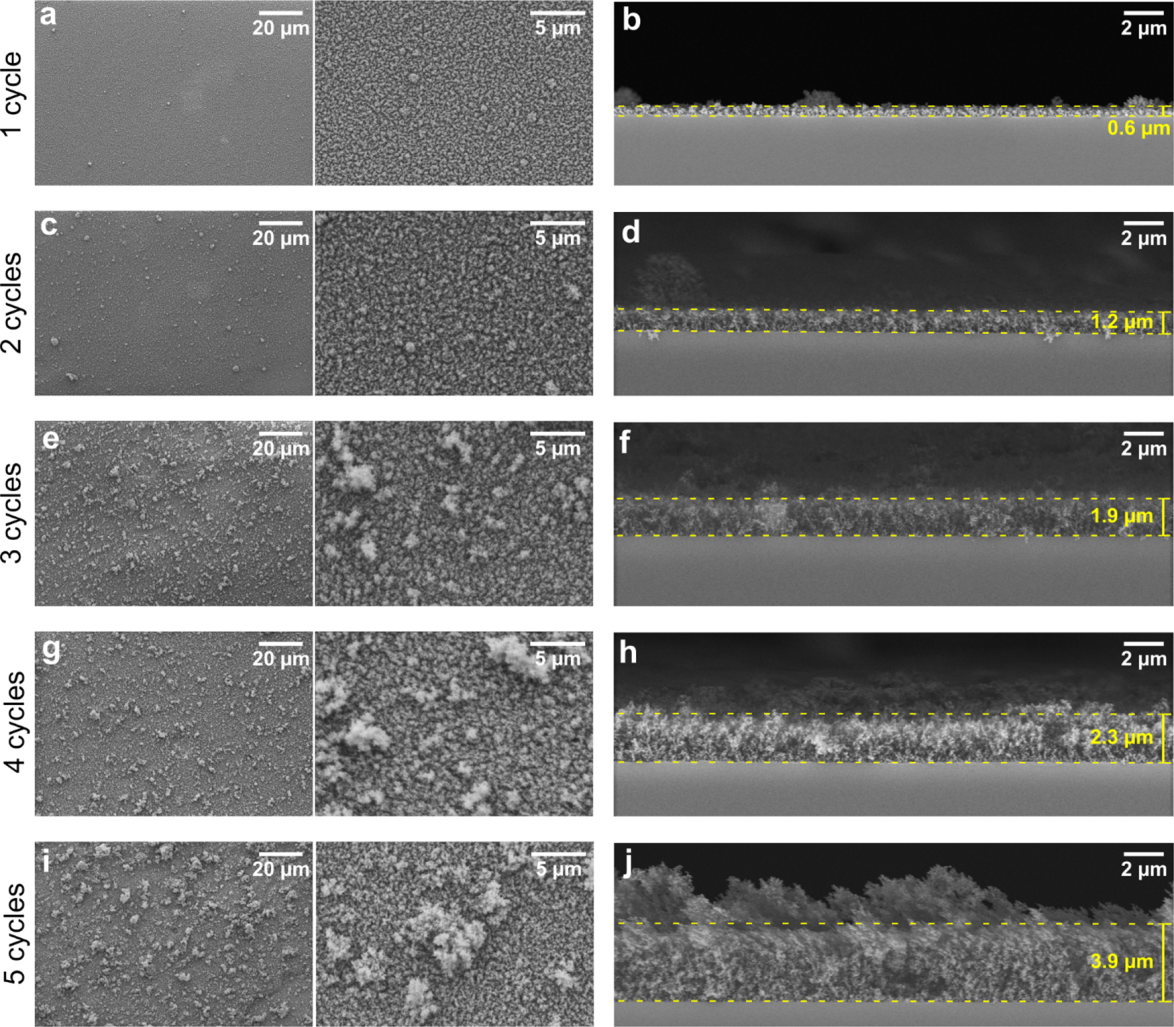
SEM images in top-view at two magnifications and in cross sections of the different coatings: (a, b) one coating cycle, (c, d) two coating cycles, (e, f) three coating cycles, (g, h) four coating cycles, and (i, j) five coating cycles. The dotted yellow lines indicate the position of the inner homogeneous layer, and the approximate thickness of this layer are reported in the cross-sectional images. N.B. some cross-section images are taken at a slight angle to achieve optimal imaging of the cross section.

Cross-sectional SEM images of the different samples show the desired increase in coating thickness with an increasing number of coating cycles (Figure 1). The homogeneous coherent parts of the coatings increase from below 1 μm for one coating cycle to approximately 4 μm for five coating cycles (Table 1). The level of hierarchical roughness and the maximum height of the protrusions also increase with an increasing number of coating cycles (Table 1).

**Table 1:** Approximate Coating Thicknesses Determined from SEM Images and Corresponding Gaseous Layer Thicknesses Determined from LSCM Images

	coating layer (μm)		gaseous layer (μm)	
sample	homogeneous part	protrusions	sessile droplet	in AFM
1 coating cycle	0.6	1.2	1	<1
2 coating cycles	1.2	3.4	3	2
3 coating cycles	1.9	3.8	4	3
4 coating cycles	2.3	4.8	5	3
5 coating cycles	3.9	7.1	7	4

### Surface Superhydrophobicity

**Figure 2:**
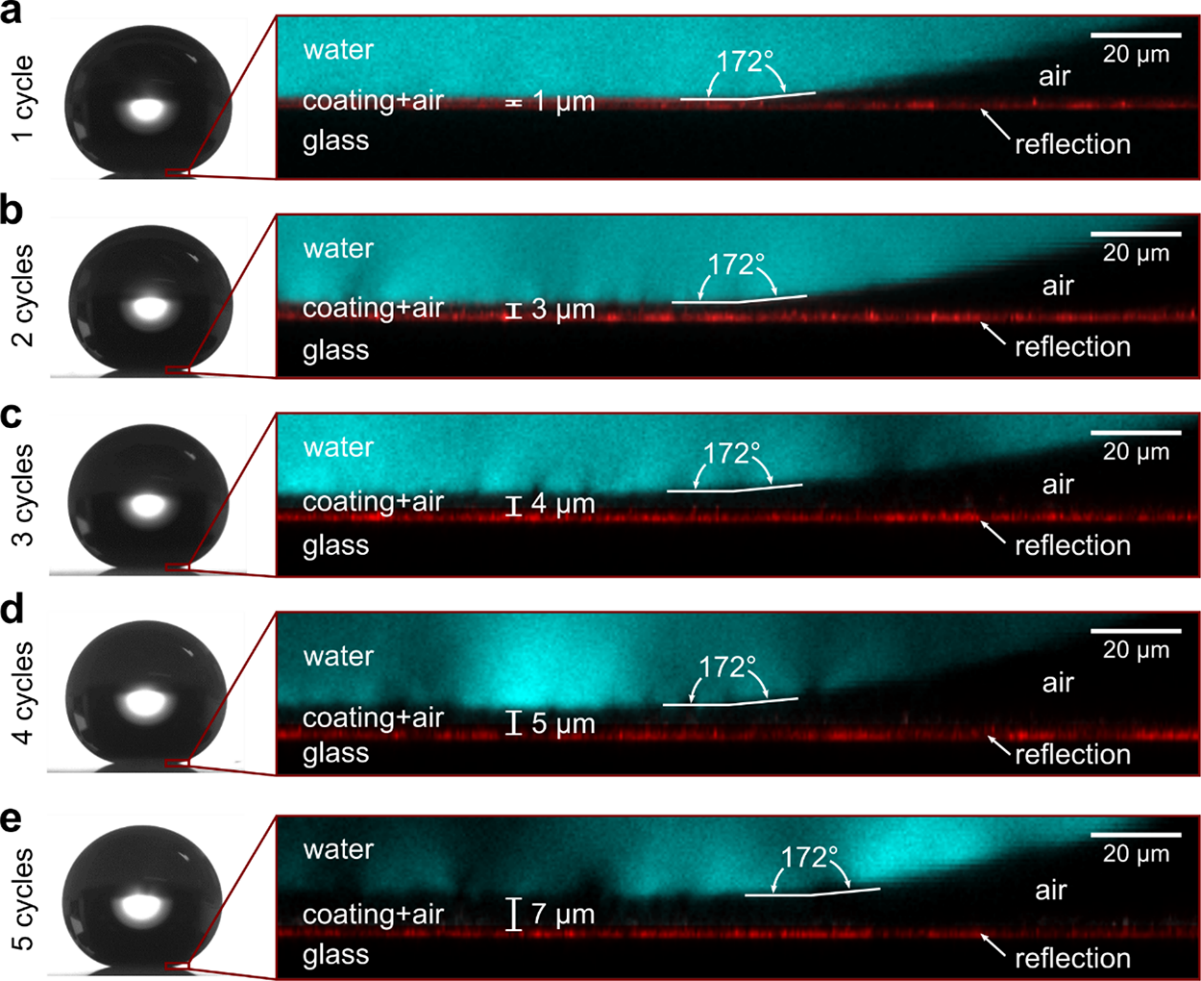
Photos showing the shape of 6 μL water drops and images using laser scanning confocal microscopy of a water drop labeled with fluorescent dye (1 mg L^–1^) (cyan color) resting on the superhydrophobic surfaces prepared with (a) one coating cycle, (b) two coating cycles, (c) three coating cycles, (d) four coating cycles, and (e) five coating cycles. The reflection from the substrate-coating interface is shown in red.

Small, 6 μL water droplets adopted an almost spherical shape when placed on all five coatings (Figure 2). We note that the local contact angles determined with the LSCM (172°) were about 10° larger than those evaluated by standard contact angle measurements (Table 2). Thus, our data support the notion that goniometer data are uncertain for high CAs (θ ≳ 150°). The reason for this is the small distance between the solid and liquid close to the contact line that hampers accurate determination in optical images.^29,30^ The wetting state is clearly of the Cassie–Baxter-type, as visualized in the confocal images. The thickness of the gaseous layers below the droplets was seen to increase with increasing number of coating cycles (Figure 2, Table 1). The thicknesses of the gaseous layers are similar to the heights of the protrusions on the coating surfaces. Thus, the water droplets are indeed suspended on top of the protrusions.

**Table 2:** Apparent Advancing (θ_adv_) and Receding (θ_rec_) Water Contact Angles and Roll-Off Angles (RA) for 10 μL Drops, Measured Using Goniometry^a^

sample	θ_adv_ (deg)	θ_rec_ (deg)	RA (deg)
1 coating cycle	163 ± 1	154 ± 4	3 ± 2
2 coating cycles	162 ± 1	156 ± 8	<1
3 coating cycles	161 ± 1	159 ± 2	<1
4 coating cycles	162 ± 1	160 ± 1	<1
5 coating cycles	161 ± 1	159 ± 1	<1

a Mean values were obtained with standard deviations.

Additionally, all coatings displayed very low roll-off angles (<5°) for 10 μL water droplets as measured with contact angle (CA) goniometry (Table 2). For samples prepared by one coating cycle, the water droplet adhered slightly to the surface, as revealed by measurable roll-off angles of 3 ± 2°. However, for samples coated using two, three, four, and five coating cycles, the roll-off angles were too low to be measured. Even on horizontal preleveled samples (without any apparent tilt angle), the droplets either rolled off as soon as the needle was detached or as soon as they became disturbed when the tilting started.

These findings and our previous work^9,14^ support the view that force measurements can distinguish and characterize ultra- and superhydrophobic systems.

### Measurements of Surface Forces and Observation of Gas Capillaries

Two-dimensional cross-sectional images through the center of the particle were recorded with LSCM during colloidal probe AFM force measurements between a hydrophobic microsphere (radius *R* = 15.6 μm as determined by SEM) and the superhydrophobic samples. These images were analyzed to obtain the shape of the capillaries.^11^ The formation of a gas capillary between the interacting surface can be seen for all five coatings (Videos S1–S5, Supporting Information).

**Figure 3:**
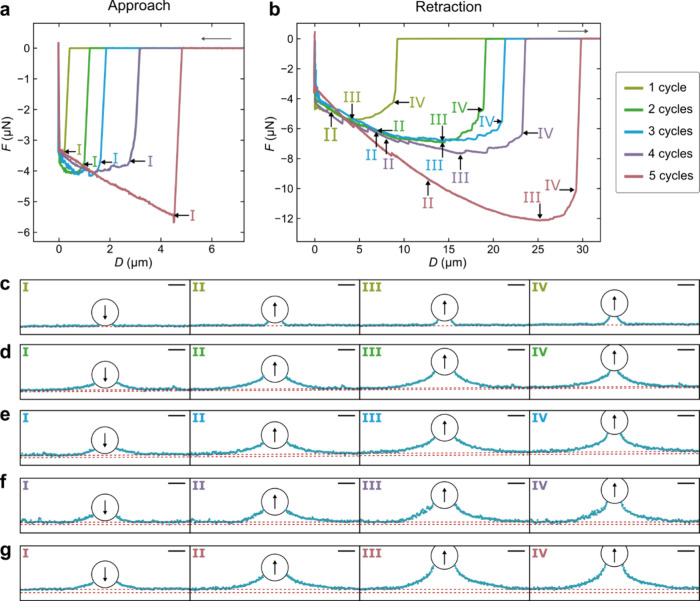
Representative force–distance curves recorded on (a) approach and (b) retraction with corresponding gas capillary menisci shapes for measurements on the different samples: (c) one coating cycle, (d) two coating cycles, (e) three coating cycles, (f) four coating cycles, and (g) five coating cycles at the different positions marked in (a) and (b). In (c)–(g), contours of the water–air interfaces are plotted in cyan, the positions of the water–gas and glass–air reflections as dashed red lines and the particle position as a black circle. Scale bar: 20 μm. The piezo expansion rate during the measurements was 0.2 μm s^–1^.

**Figure 4:**
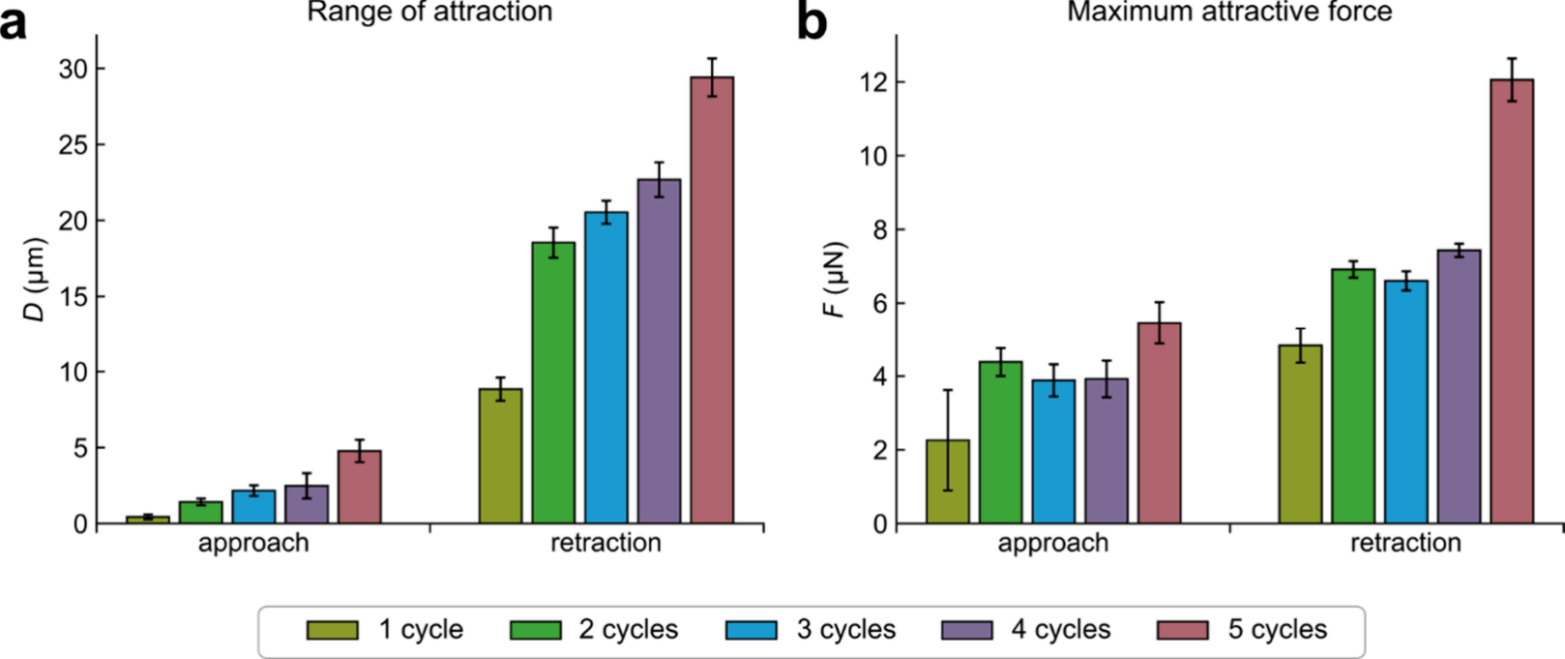
(a) Range of attraction and (b) maximum values of the attractive force on approach and retraction. Error bars show standard deviations.

During a force measurement, the hydrophobic particle first approaches the superhydrophobic surface (Figure 3a). At large separations, the force is zero. Subsequently, at sufficiently small separation, a strongly attractive force (defined as negative) suddenly appears. The distance depends on the thickness of the coating. Corresponding confocal images at this point show the sudden appearance of a bridging gas capillary (Figure 3a, point I). The separation at which the attractive force appears is called the range of attraction observed on approach, which clearly increases with an increasing number of coating cycles (Figures 3a and 4a). When the colloidal probe came into contact with the superhydrophobic coating at zero separation, the cantilever is retracted (Figure 3b). The attractive force persists as the separation distance increases during separation. At this stage, the attractive force increases, and at the same time, the gas capillary spreads on the superhydrophobic surface (Figure 3, point II) until a maximum attractive force is reached (Figure 3b, point III). The maximum attractive force observed on retraction is smallest for one coating cycle. It increases in accordance with the number of coating cycles, reaching the largest value for the sample having five coating cycles (Figures 3b and 4b). After the attractive force maximum is reached, the attractive force then gradually decreases as retraction proceeds until the point at which the capillary suddenly ruptures (Figure 3, point IV) and the force immediately returns to zero. Generally, the range of the attraction during separation also increases with the coating thickness (number of coating cycles) (Figures 3b and 4a).

### Gas Capillary Characterization and Development during Retraction

**Figure 5:**
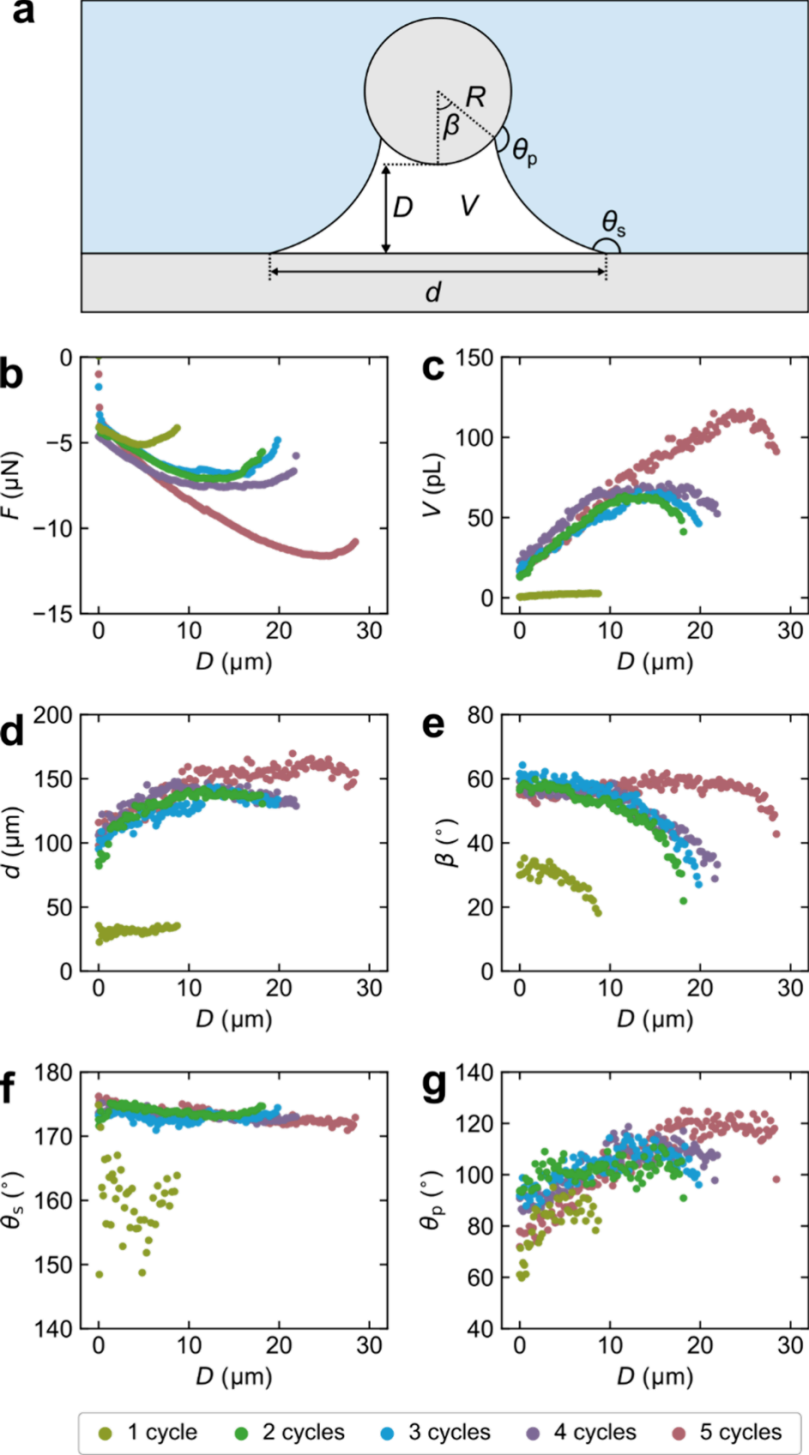
(a) Schematic illustration of a gas capillary (volume *V*) between a spherical particle of radius *R* and a flat surface at separation distance *D*, with the diameter of the dewetted area on the flat surface *d*, the angle of the dewetted area on the particle β, and the contact angles of the flat surface θ_s_ and particle θ_p_ at the gas–liquid interfaces. Diagrams of (b) the measured force, *F*, (c) *V*, (d) *d*, (e) β, (f) θ_s_, and (g) θ_p_ as a function of *D* for representative measurements on the samples prepared with different coating cycles.

**Figure 6:**
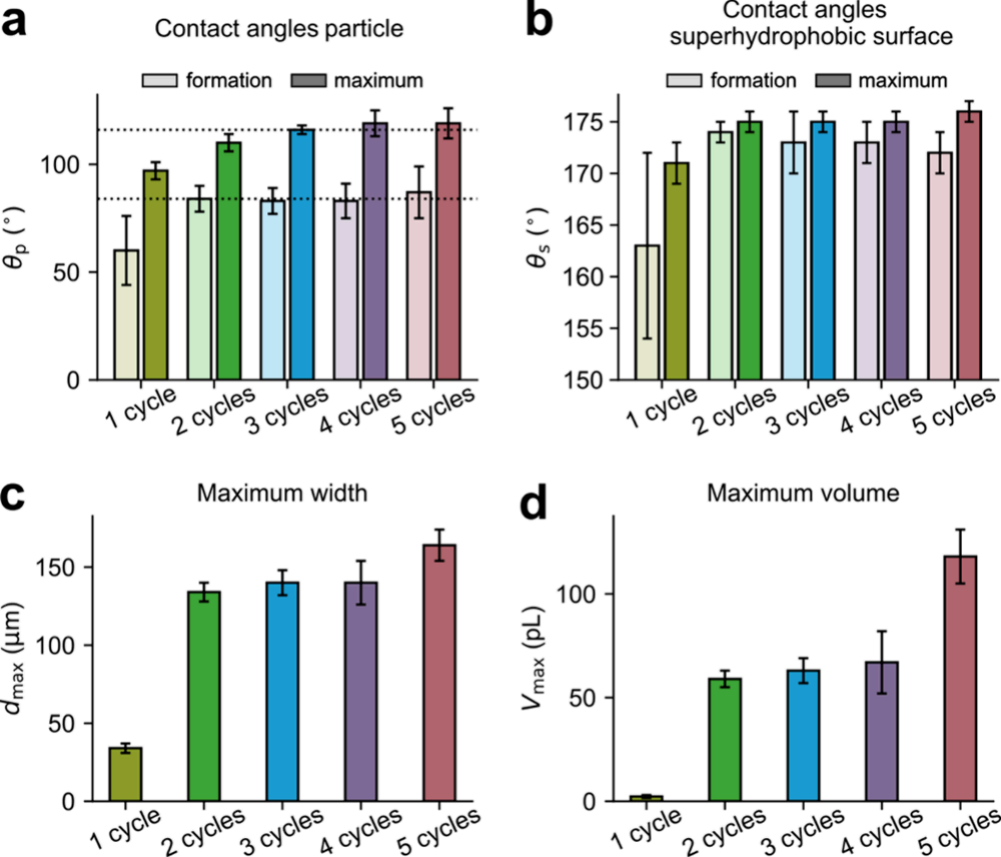
Data from capillary images during force measurements: capillary contact angles at formation (= receding contact angle, light color) and maximum contact angle (= advancing contact angle, darker color) on (a) the particle θ_p_ and (b) the superhydrophobic surface θ_s_, (c) maximum value of the capillary width on the superhydrophobic surface *d*_max_, and (d) maximum value of the capillary volume *V*_max_. The horizontal dotted lines in (a) are the receding (θ_rec_ = 84°) and advancing (θ_adv_ = 116°) contact angles measured on a flat surface having similar chemistry. Error bars show the standard deviations.

From the capillary meniscus shape obtained from confocal images, we determined the following: (i) the capillary volume *V*, (ii) the diameter of the dewetted area on the superhydrophobic surface *d*, (iii) the angle defining the dewetted area on the particle β, and (iv) the capillary CAs at the gas–water interface on the colloidal probe θ_p_ and superhydrophobic coating surface θ_s_ (Figure 5a). Gas capillaries observed on the five different coatings follow a similar development during retraction of the particle (Figure 5). For the main part of the retraction, when the attractive force increases (Figure 5b), the gas capillaries grow in volume (Figure 5c) on all five coatings. However, gas capillaries observed for samples prepared using one coating cycle are considerably smaller, both initially and at maximum size, than those observed on the other four coatings. As the capillary volume increases, the gas capillaries spread on the superhydrophobic surfaces with a low degree of pinning of the three-phase contact line (TPCL) (Figure 5d). In contrast, the TPCL is typically pinned on the particle surface until the maximum attractive force is reached (Figure 5e). The capillary CA on the particle increases as long as the TPCL is pinned and reaches a maximum value, 110–120°, when the TPCL starts to recede on the particle surface (Figure 5e,g). Once the pinning force has been overcome on the particle, the capillary rapidly breaks. The maximum CA on the particle is similar to the advancing contact angle of 116° determined on a flat fluorosilanized microscope glass slide (Figure 6a), i.e., with a similar surface chemistry as on the particle. Thus, the wetting situation on the particle, rather than on the superhydrophobic surface, determines the stability of the capillary, and the capillary is disrupted when the dynamic CA on the particle approaches the advancing CA.

In general, as mentioned above, we observe much smaller capillaries on the sample prepared using one coating cycle as compared to the thicker coatings (Figure 6c,d), suggesting that the amount of accessible gas present in the surface voids under the Cassie–Baxter state at the surface limits capillary growth and that less gas exists in the case of the samples prepared using only a single coating cycle. For this single coating cycle case, the capillaries are smaller than for the other samples also at the formation stage (Figure 3c, point I), with smaller initial *d* and β as compared to those of the other four coatings (Figure 5d,e). Thus, the less rough surface achieved by one coating cycle compared to multicycle coatings (Figure 1) limits the initial capillary size and capillary growth during the separation process. It is natural to assign this to less available gas in the coating combined with increased difficulty of gas diffusion to the capillary. This difficulty, in turn, results from the fact that gas that leaves the coating must be replaced with water to avoid a large under pressure, and thus in small pores, solid–vapor contacts in the coating will be replaced by solid–water contacts. This is unfavorable when the local contact angle is above 90°, as in our case. Furthermore, the increase in the capillary volume with a number of coating cycles suggests that any dissolved gas in the water, which, by definition, is constant, or direct vaporization of water, contributes minimally in the case of these greatly extended attractive forces. This supports the conclusion that the capillary volume achievable depends on the existing gas contained in the surface layer prior to particle–surface contact.

In the confocal images recorded during force measurements, we notice that the thickness of the gaseous layer at the superhydrophobic surfaces under complete submersion is thinner than that observed for a water droplet resting on the surfaces (Figure 2, Table 1) and shows closer correlation with the thickness of the homogeneous part of the coating, rather than with the thickness of the coating protrusions. For the sample prepared by one coating cycle, the gaseous layer during force measurements is even too thin to be resolved in the confocal images (<1 μm). One reason for the observed thinner layer could be that the water partially penetrates the coating layers at the same time as gas in the coating dissolves into water. Another reason is due to trapping of water between the probe and the coating in the AFM, where the water droplet is initially pressed against the superhydrophobic coating. This causes water to flow along the surface until pressure equilibrium with the surrounding is established. However, we were still able to see a small increase in thickness of the gaseous layer during force measurements with increasing number of coating cycles. The small difference in air layer thickness can, therefore, explain the small differences observed in capillary size and attractive forces between samples prepared using two, three, and four coating cycles. The reason why the gas capillaries on the sample with five coating cycles are observed to grow considerably larger is suggested to be due to the disproportionately increased surface roughness. Thus, the total amount of accessible gas in and on the coating is determined by its thickness, porosity, and surface roughness. The volume ratio of gas to solid (coating) will, for a given thickness, increase with increasing surface roughness (or porosity), and this may also allow capillaries to grow larger.

The microscopic CAs of the capillary on the particle and superhydrophobic surface can be compared with the macroscopic CAs on each surface. The receding CA (θ_rec_) is expected to be observed at capillary formation as the water needs to recede when the particle and superhydrophobic surfaces are dewetted. Further, the advancing CA (θ_adv_) is expected to be equal to the largest observed contact angle during retraction, which is observed when the water is advancing over the surfaces and the dewetted gas-filled areas become wetted again at surface extremities contact, returning to the Cassie–Baxter state. For CAs on the particle, there is good agreement between the macroscopic CAs measured on a flat chemically similar surface (θ_rec_ = 84° and θ_adv_ = 116°) and CAs for capillaries on samples prepared using two, three, four, and five coating cycles (Figure 6a). However, for the sample prepared with one coating cycle, the capillary CAs on the particle during retraction are lower in the initial and final stages. As the shape of the capillary meniscus is expected to be determined by the surface tension and capillary pressure (discussed below), the observed lower CAs for one coating layer might suggest that the contact angle changes to minimize the total free energy with a relatively small gas capillary. A higher probability of pinning, as indicated by the higher roll-off angle, may also be the case.

The capillary CAs observed on the superhydrophobic surfaces are larger as compared to the macroscopic θ_adv_ and θ_rec_ values as measured for droplets using goniometry (Table 2). For samples prepared using two, three, four, and five coating cycles, the CAs at the hydrophobic surface at the point of capillary formation are approximately θ_s_ ≈ 173° and the largest observed CA during separation is approximately θ_s_ ≈ 175° (Figure 6b). These values are in closer agreement with CAs observed with LSCM (172°, Figure 2). Again, the CA observed for samples prepared using one coating cycle is lower, θ_s_ = 163° at capillary formation, and the largest observed CA on separation is θ_s_ = 171°.

### Calculations of Interactional Forces from Capillary Shape and Comparison with Measurements

**Figure 7:**
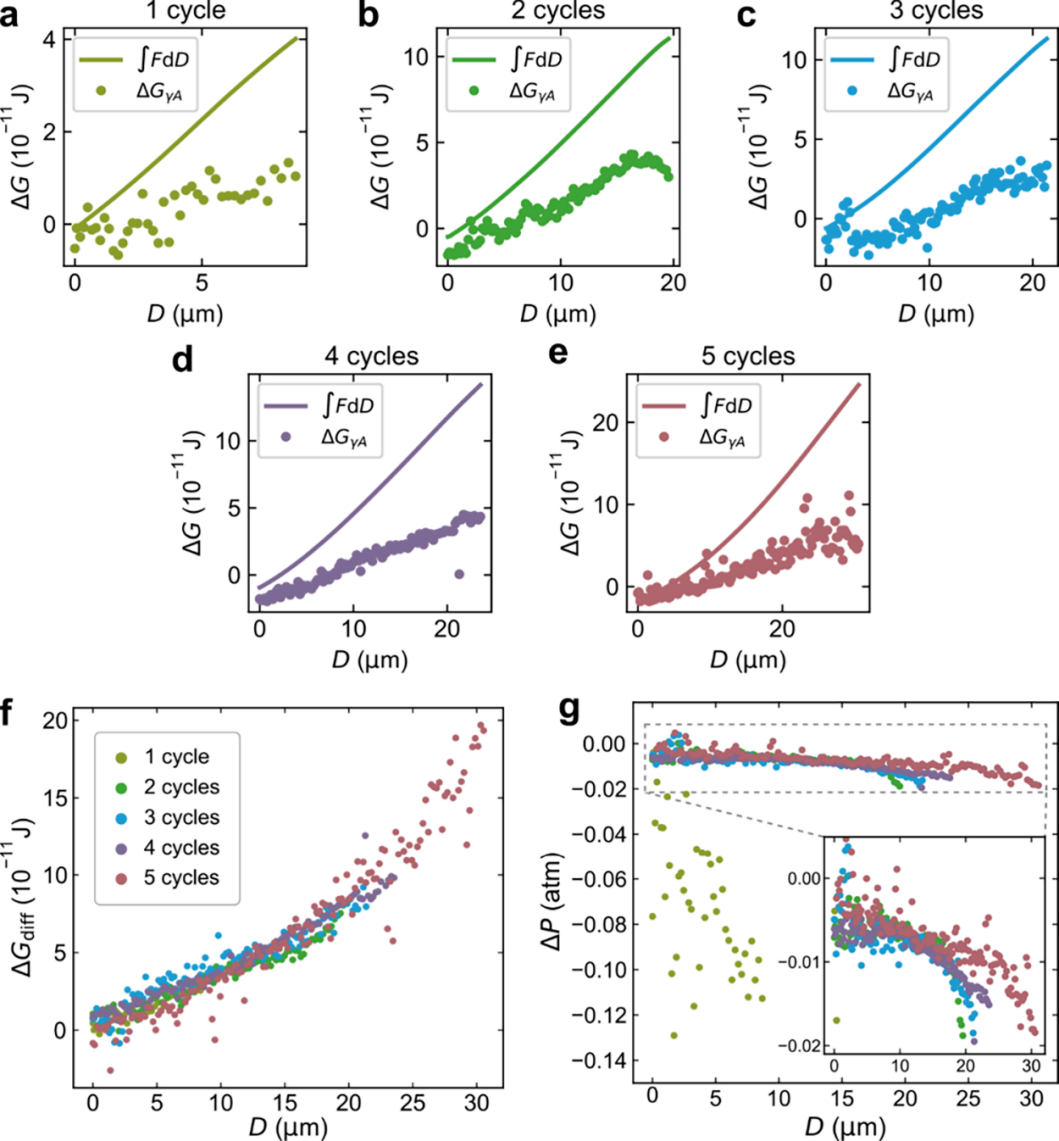
Change in free energy due to formation of gas capillaries assessed from the integral of the force–distance curve ∫*F* d*D* (solid lines) compared to the surface tension-area work Δ*G*_γ*A*_ (symbols) during retraction for measurements on the different coatings: (a) one coating cycle, (b) two coating cycles, (c) three coating cycles, (d) four coating cycles, and (e) 5 coating cycles, (f) the difference Δ*G*_diff_ between ∫*F* d*D* and Δ*G*_γ*A*_ in (a–e), and (g) the capillary pressure Δ*P* calculated from Δ*G*_diff_ as , assuming Δ*PV* being the sole contribution to Δ*G*_diff_.

The capillary force can be estimated by considering the free energy change resulting from the formation of the capillary. The total surface free energy change, related to the dynamic interfaces constituting a total area *A*, includes contributions from surface tension γ, the capillary pressure Δ*P*, and properties at the TPCLs. The surface tension partial contribution Δ*G*_γ*A*_ is given by the increase in free energy associated with creating the gas–liquid interface, and the free energy change due to dewetting of the particle and superhydrophobic coating:
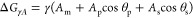
1where *A*_m_ is the surface area of the capillary meniscus gas–liquid interface. *A*_p_ and *A*_s_ are the dewetted areas on the particle and the superhydrophobic surface, respectively, and can be evaluated from confocal images together with θ_p_ and θ_s_. The calculated values of Δ*G*_γ*A*_ can be compared to the measured force by integrating the measured force–distance curve ∫*F* d*D*. The initial energy at zero distance is calculated by the integration of the approach force curve and added to the integral of the separation force curve. The capillary is thermodynamically stable as long as the change in free energy is negative (Δ*G*_γ*A*_ < 0), which is only the case at small separations (Figure 7a–e). At large separations, when Δ*G*_γ*A*_ becomes positive, the gas capillary is metastable. It remains in a metastable state until it ruptures, which occurs when the energy barrier for rupturing becomes sufficiently small.^31^

As expected, we see a difference between the measured ∫*F* d*D* and the calculated Δ*G*_γ*A*_ for a large *D* in all five cases (Figure 7a–e). This is because we did not include contributions due to pressure–volume work, Δ*PV*, or contributions from the TPCLs when expressing the equilibrium in terms of Δ*G*_γ*A*_ alone. We note that the difference
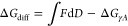
2is increasing with separation similarly for all five coatings (Figure 7f).

The individual contributions from Δ*PV* and, especially, TPCLs cannot be easily evaluated from the present data since the tortuous and deformed TPCL path could not be determined from our confocal images.^29,30^ If Δ*G*_diff_ is restricted to being caused by Δ*PV* alone, the capillary pressure can be estimated by
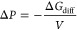
3

For the samples prepared by two, three, four, and five coating cycles, such analysis shows that only a limited capillary under pressure (<0.02 atm) is needed to account for the observed difference, while for the sample prepared with one coating cycle, it is considerably higher (Figure 7g). This is likely a result of the limited amount of gas that can be transported to the capillary from the superhydrophobic surface of the single coating layer. It seems reasonable that we have a slight under pressure in the capillary in our dynamic measurements as long as the capillary grows and gas flows into the capillary. We note, however, that the data in Figure 7g suggest that the under pressure increases somewhat in magnitude at large distances as the capillary volume shrinks and gas flows out. This seems unreasonable since it suggests that gas moves from the low-pressure capillary region to a region with a higher pressure (the gaseous layer and the water phase). The observation rather implies that free energy contributions arising from TPCL effects are important,^6,16^ particularly when the gas capillary shrinks in size. Additionally, such effects may play a larger role for the sample generated using only one coating cycle, where the contact angle hysteresis is greater than that of the multicycle coated samples.

## Conclusions

We investigated the effects of the amount of trapped gas associated with a nanostructured surface layer exhibiting superhydrophobicity when immersed in water during force–distance measurements using a hydrophobic microsphere probe. Long-range attractive capillary forces were detected, related to the formation of bridging gas capillaries within the surrounding water medium. An increased thickness of the nanostructured surface layer, devised by applying further nanoparticles in cycles through a liquid flame spray, increased the range of the attractive forces and the size of the capillaries. Together with the microscope observation that the surface structural voidage also increased with layer thickness, these results suggest that capillary formation and growth, and hence the range and magnitude of the interaction force during retraction of the probe, are strongly affected by the amount of accessible gas in the surface layer. This conclusion was underlined by the strikingly reduced size of the capillaries in the case of the thinnest coating layer. Furthermore, the limiting factor of available gas on capillary growth allows us to deduce that dissolved gas in the water or direct vaporization of water, or both, is insufficient alone to establish the very long-range attraction observed when one of the interacting surfaces is superhydrophobic and in the Cassie–Baxter state. Comparison between measured force and calculated free energy change, derived primarily from capillary interactions, suggested a limited under pressure in the gas capillary but also suggested that three-phase contact line effects may be of importance. To evaluate quantitatively the three-phase contact line effects remains a challenge.

## Supporting Information Available

Videos S1 to S5 showing the formation and rupture of a gas capillary between the probe and surface for the five coatings using laser scanning confocal microscopy (AVI)

## Supplementary Material

la3c03709_si_001.avi

la3c03709_si_002.avi

la3c03709_si_003.avi

la3c03709_si_004.avi

la3c03709_si_005.avi
